# Predisposing Factors and Incidence of Venous Thromboembolism among Hospitalized Patients with Sickle Cell Disease

**DOI:** 10.3390/jcm12206498

**Published:** 2023-10-12

**Authors:** Mohammed S. Ziyadah, Eman M. Mansory, Hatem M. Alahwal, Salem M. Bahashwan, Abdullah T. Almohammadi, Osman O. Radhwi, Talal Alghamdi, Shahida A. Khan, Majed N. Almashjary, Ahmed S. Barefah

**Affiliations:** 1 Department of Internal Medicine, Faculty of Medicine, King Abdulaziz University Hospital, King Abdulaziz University, Jeddah 21589, Saudi Arabia; ziyadahmo@mngha.med.sa (M.S.Z.); talal.alghamdi@medportal.ca (T.A.); 2 Hematology Department, Faculty of Medicine, King Abdulaziz University Hospital, King Abdulaziz University, Jeddah 21589, Saudi Arabia; emmansory@kau.edu.sa (E.M.M.); halahwal@kau.edu.sa (H.M.A.); smbahashwan1@kau.edu.sa (S.M.B.); atalmohammade@kau.edu.sa (A.T.A.); oradhwi@kau.edu.sa (O.O.R.); 3 Hematology Research Unit, King Fahd Medical Research Center, King Abdulaziz University, Jeddah 21589, Saudi Arabia; malmashjary@kau.edu.sa; 4 Applied Medical Nutrition Group, King Fahd Medical Research Center, King Abdulaziz University, Jeddah 21589, Saudi Arabia; sakhan01@kau.edu.sa; 5 Department of Medical Laboratory Sciences, Faculty of Applied Medical Sciences, King Abdulaziz University, Jeddah 21589, Saudi Arabia

**Keywords:** sickle cell disease, venous thromboembolic events, deep vein thrombosis, pulmonary embolism

## Abstract

Though patients with sickle cell disease (SCD) are at risk of developing venous thromboembolism (VTE), clear estimates of its incidence and predisposing factors in hospitalized SCD patients are not available. Therefore, this issue was addressed to facilitate an early diagnosis and initiate appropriate prophylactic and treatment strategies. A retrospective observational study was conducted on patients with SCD who were admitted to an academic center in Saudi Arabia over a 10-year period. We identified 1054 admissions of 394 patients with SCD. Of the 3% of patients identified with VTE, 50% experienced pulmonary embolism (PE), 34.3% exhibited deep vein thrombosis (DVT), 6.3% exhibited cerebral vein thrombosis, and 9.4% showed other forms of VTE. In pregnant SCD patients, 6.4% developed a VTE event during their hospital admission. Of the risk factors, high white blood cell count, length of stay, and presence of any additional risk factor for VTE was associated significantly with higher risk of VTE. In our study, this risk seems to be much lower, which is likely attributed to the use of VTE prophylactic strategies implemented in our center. Nevertheless, further studies are needed to establish the ideal prophylactic strategy in patients with SCD.

## 1. Introduction

SCD is a widespread hematological disorder that has garnered significant attention due to its global prevalence and the challenges it presents. This disease primarily affects hemoglobin molecules, leading to alterations in the shape and function of red blood cells [1]. It is inherited in an autosomal recessive manner, indicating that both parents must carry the gene for a child to be affected. The underlying pathophysiology of SCD is attributed to a point mutation in the β-globin gene. This mutation results in the replacement of glutamic acid with valine, subsequently causing the erythrocytes to adopt a sickle shape, which can lead to various complications [1]. The disease is inherited either in the homozygous (HbSS) or heterozygous form, including sickle cell trait where only one allele harbors the mutation (HbAS), HbS with hemoglobin C (HbSC), and S-beta0 and b+ thalassemia (HbSβ° and HbSβ^+^, respectively) [2]. In the context of global prevalence, Saudi Arabia stands out with a notably high incidence of SCD. Specifically, regions in the eastern and southwestern parts of the country report a prevalence rate of approximately 2.6% [3,4].

The clinical implications of SCD are vast and multifaceted. Patients diagnosed with SCD inherently possess a hypercoagulable state, which significantly elevates their susceptibility to thromboembolic events, subsequently increasing morbidity and mortality risks [5,6]. This heightened risk has been corroborated by studies from diverse geographical regions, including Oman, Saudi Arabia, and the United States. These studies have consistently reported a substantial prevalence of venous thromboembolism (VTE) in SCD patients, with rates fluctuating between 8 and 25% [6,7,8,9,10]. The etiology behind this elevated VTE incidence in SCD patients is multifactorial. Factors such as chronic inflammation, which activates the coagulation cascade, tissue damage from vaso-occlusive crises-induced ischemia, surgical interventions such as splenectomy, reduced spleen function (hyposplenia), prolonged immobility during hospitalizations, and the employment of central venous catheters collectively contribute to this risk [10,11]. The clinical ramifications of VTE in SCD patients are severe, with a marked increase in mortality rates and a heightened demand for intensive care [8]. Compounding these challenges, the typical clinical manifestations of SCD often overshadow VTE’s signs and symptoms, posing significant hurdles in timely diagnosis and treatment initiation [12].

Considering these challenges and to bridge the knowledge gap, a retrospective study was initiated. This research endeavor aims to delve deeper into the incidence of VTE in hospitalized SCD patients and elucidate the predisposing factors associated with it. Through this study, we aspire to quantify the burden of VTE in SCD during hospitalization and furnish comprehensive data that could guide the medical community in devising effective prophylactic, diagnostic, and therapeutic strategies.

## 2. Materials and Methods

### 2.1. Participants and Study Design

This is a retrospective observational analytical cohort study on hospitalized SCD patients to the different wards in King Abdulaziz University Hospital (KAUH), regardless of the reason for admission from the period of 1 January 2010 to 15 November 2020. The sociodemographic data, type of SCD, complete blood count (CBC), comorbidities, and medical history were obtained and reviewed from the hospital information system at KAUH. The study’s inclusion criteria were patients with SCD above the age of 18 years, hospitalized for a period of more than 24 h. The thrombotic event registered during the first time of hospitalization was taken for analysis in cases of multiple hospitalizations of patients with VTE. Patients under the age of 18 years and those admitted only for less than 24 h were excluded from the study.

### 2.2. Participants Clinical and Laboratory Information

In this retrospective study, demographic data, including the patient’s age and gender, were collected. Detailed data on the length of hospital stay, reason and number of admissions, previous surgery and/or trauma, immobility, use of hormonal therapy, pregnancy, and the presence of intravenous catheter were obtained from the hospital electronic system. Results of the laboratory tests of the admitted patients such as hemoglobin electrophoresis and complete blood count (CBC) at day ‘0′ of admission were also collected. The risk factors taken into consideration in this study were white cell count, platelet count, and hemoglobin values, in addition to the effect of using antithrombotic, nonsteroidal anti-inflammatory drugs (NSAIDs), and hydroxyurea (HU) on the incidence of VTE.

### 2.3. Primary Outcome

The primary outcome was the presence of a confirmed VTE event during admission which is supported by ultrasound, magnetic resonance imaging (MRI), or computed tomography (CT) angiography reports. Different VTE types, such as deep venous thromboembolism (DVT), pulmonary embolism (PE), and cerebral vein thrombosis, were included and the timing of the VTE was recorded (1–7 days post admission vs. >7 days post admission).

This study was conducted at KAUH and was approved by the Biomedical Ethics at the Faculty of Medicine and King Abdulaziz University Hospital (No. 511-20). The study followed the STROBE guidelines for reporting data.

### 2.4. Data Analysis

Fisher’s exact test was used for the categorical variables and presented as frequency with proportions to compare baseline and disease-related characteristics. Continuous data were reported as means with ranges. The Wilcoxon rank-sum test was utilized to compare the two groups. Values of *p* < 0.05 were considered statistically significant. Variables demonstrating statistical significance in univariate analysis were included in a multivariate analysis using a logistic regression model. The unit of analysis was hospitalization events unless otherwise specified. All statistical analysis was performed using Stata (ver. 17.0).

## 3. Results

### 3.1. Patients’ Clinical Information and VTE Risk Factors

**Figure 1:**
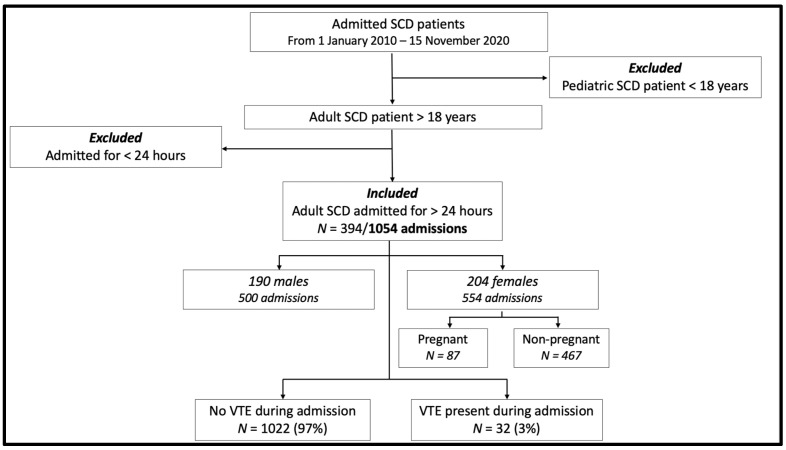
Flowchart of the selection process for admitted SCD patients from 1 January 2010 to 15 November 2020. Initial patient pool consisted of both pediatric (<18 years) and adult (>18 years) SCD patients. Pediatric patients and adults admitted for less than 24 h were excluded. The final cohort included 394 out of 1054 admissions, with 190 males accounting for 500 admissions and 204 females accounting for 554 admissions. Of these, 1022 (97%) had no VTE during admission, while 32 (3%) presented with VTE during admission.

The study enrolled 394 patients with SCD, 190 males and 204 females. Combined together, a total number of 1054 admissions took place from the period of January 2010 to November 2020. Of these admissions, there were 500 adult males and 554 adult females, respectively, with a mean age of 27.4 years. The flowchart in Figure 1 visually represents the patient selection process, detailing the inclusion and exclusion criteria and the distribution of the patients based on gender and VTE occurrence during their hospital stay.

Table 1 shows the patient demographics, along with the reasons for admission and potential risk factors for VTE. Most of the admissions in both sexes (75.9%) were for SCD-related complications such as crises (hemolytic, aplastic, or painful crisis), stroke, cholecystitis, pneumonia, or acute chest syndrome. Seven patients were admitted experiencing a VTE event on admission (0.7%), while the rest were admitted for reasons (*n* = 254, 24.1%) other than the above-mentioned reasons. Most of the admitted cases (*n* = 817, 77.5%) had no known VTE risk factors, other than being SCD patients, while the remaining 22.5% (*n* = 237) were admitted with known VTE risk factors. The most common risk factors were pregnancy (*n* = 87, 8.3%) and surgery (*n* = 63, 6%) and others as listed in Table 1.

Among the 554 admissions in females with SCD, 87 admission (15.7%) were for pregnant patients at various stages of pregnancy, as shown in Table 1. Interestingly, five of these admissions (6.4%) were complicated by a VTE event (three PEs and two DVTs). Of the admissions during pregnancy, 61 admissions (70.1%) were on prophylactic-dose anticoagulation during their admission, while 4 (4.9%) were on intermediate/full-dose anticoagulation and 22 (25.3%) were not on anticoagulation therapy.

### 3.2. Venous Thrombotic Events (VTE) and Types of Treatments Given

The presence, type, timing of VTE events, and the medical treatment given to the admitted SCD patients were assessed as shown in Table 2. Out of the 1054 SCD hospitalizations, 32 admissions (3%) were associated with a VTE event, 50% of which were PE cases, 34.3% had DVT, and 6.3% had cerebral vein thrombosis. Of the patients who had a VTE event, VTE was the reason for admission in 21.9%, while 56.2% experienced it within 7 days of their admission and another 21.9% had VTE after 7 days from admission. Therefore, of the 32 (3%) patients with VTE, 25 of them experienced it after admission, which amounts to 2.34% of the total number of SCD patients.

Around 75.7% of the admitted patients received antithrombotic treatments during their hospital stay, whether prophylactic or therapeutic. Prophylactic low-molecular-weight heparin (LMWH) was administered to 63%, while 4.8% received therapeutic LMWH. Prophylactic unfractionated heparin was given to 2.3% (*n* = 24) of the admitted SCD patients, while 0.2% (*n* = 2) were given therapeutic unfractionated heparin. Reasons for full-dose anticoagulation included the following: 20 patients with known history of VTE events outside of the included hospital admissions, 2 patients with history of non-valvular atrial fibrillation, and 2 patients with valvular heart disease secondary to rheumatic heart disease. The remaining admissions on full-dose anticoagulation were for the purpose of managing the intensity of their VOC as a local practice influenced by a previous RCT [13]. Hydroxyurea, the only available and approved anti-sickling agent at the time of the study, was administered to 42% (*n* = 443), whereas NSAIDs were prescribed to 50.9% of the admitted patients with SCD.

### 3.3. Association between the Risk Factors and VTE Incidence

A multivariate regression analysis was conducted to examine the association between the risk factors and VTE incidence, as shown in Table 3. Patients who were on anticoagulation of any form had fewer VTE events (OR: 0.4; 95% CI: 0.192 to 0.850). The incidence of VTE was associated proportionally with the length of the stay (OR: 1.01; 95% CI: 1.00 to 1.02) and WBC count (OR: 1.04; 95% CI: 1.01 to 1.07). None of the other considered factors were found to be statistically significant in this analysis.

## 4. Discussion

Our study, conducted over a decade at a prominent academic center in Saudi Arabia, revealed that a VTE event complicated 3% of hospitalizations involving patients with SCD. Notably, these events predominantly manifested as pulmonary embolism (PE) events (50%), followed by deep venous thromboembolism (DVT) at 32.1%, cerebral vein thrombosis at 3.6%, and other VTE forms accounting for 14.3%. A significant observation was that over half (53.1%) of these VTE events occurred within the first week of admission. Our unadjusted multivariate regression analysis indicated that prophylactic anticoagulation was associated with a reduced incidence of VTE events (OR: 0.4; 95% CI: 0.192 to 0.850). Conversely, an extended hospital stay and elevated WBC count were identified as potential risk factors, albeit with only marginally increased odds.

Comparatively, two substantial cohort studies, encompassing 6237 and 1523 patients, respectively, reported that, by age 40, the cumulative VTE incidence among SCD patients ranged between 11.3% and 12.5% [10,14]. A systematic review, which included diverse study designs and patient groups, estimated a significantly elevated VTE risk in SCD or SCT patients, with even higher odds during pregnancy and postpartum periods [15]. These studies, however, did not exclusively focus on hospitalized patients, highlighting the unique contribution of our research.

The multifaceted pathophysiology underlying the heightened VTE risk in SCD patients has been the subject of extensive research [16]. Factors such as red cell dehydration, increased red cell density due to HbS polymerization, and subsequent alterations in the hemostatic system contribute to a persistent hypercoagulable state in SCD patients [11]. Numerous molecular and cellular mechanisms, including endothelial activation, elevated clotting factor levels, and increased microparticle generation, further corroborate the view of SCD as an inherent thrombophilia [13,17,18,19,20,21,22].

In the general population, hospitalization is one of the most important risk factors for VTE, though the incidence varies considerably between different institutions and nations. Some reports suggest that the hospitalized patients’ risk is 100 times higher when compared to the general population [23]. When it comes to SCD patients, in a study conducted in the United States with 1,804,000 patients hospitalized with SCD from 1979 through 2003, a discharge diagnosis of PE was made in 0.5% patients and DVT in 0.61%. Only PE events were found to be higher when compared to African American controls. Moreover, patients with SCD and VTE events were younger than hospitalized African American patients with VTE (mean age of 28 vs. 57) [24]. It is likely that thromboprophylaxis practices contributed to those rates. In our study, about 75% of patients were on some form of anticoagulation and we presume that the VTE rates would have been higher without such a practice.

In this cohort study, pulmonary embolism (PE) was observed to be the most prevalent type of VTE event, aligning with previous findings in SCD patients [10,14,24]. Notably, thrombosis in situ in large pulmonary vessels has been reported in approximately 20% of patients diagnosed with ACS [25]. This prompts the question of whether thrombosis in situ is more prevalent in SCD patients due to the underlying vascular inflammation and nitrous oxide depletion [26]. Further investigations are essential to delve deeper into this phenomenon.

Regarding admissions of pregnant females, 6.4% experienced complications with a VTE event, even though around 75% were on some form of anticoagulation during their admission. This risk is notably higher compared to the general VTE risk in pregnant females, which stands at 0.2% [27]. A retrospective review of 139 pregnant SCD patients reported a VTE incidence rate of 17% [28]. Furthermore, out of 212 delivery hospitalizations in African American women with SCD, 6 patients (2.8%) experienced complications with a VTE [29]. A national Medicaid data analysis encompassing 6388 deliveries in SCD patients indicated a VTE event rate of 11.3%, in contrast to 1.2% in controls [30]. These findings collectively suggest a tenfold increase in VTE risk among pregnant SCD patients compared to the general pregnant population. This underscores the need for further research to determine appropriate preventive measures for this demographic. Current guidelines recommend thromboprophylaxis only during antenatal hospital admissions [31], but a more comprehensive approach to risk stratification for prophylaxis outside of hospital admissions is warranted.

Several studies have explored potential risk factors contributing to VTE in SCD. A comprehensive study in 2020, which included 1193 pediatric and adult SCD patients, identified factors such as a lower estimated glomerular filtration rate, hydroxyurea use, HbSS/Sβ0 genotype, elevated white blood cell (WBC) counts, and Hb level as independent risk factors associated with VTE [32]. A 2021 study from Oman on 102 SCA patients (68 with VTE and 34 controls) highlighted high WBC count, serum lactate dehydrogenase (LDH), bilirubin, C-reactive protein (CRP), and low hemoglobin and hemoglobin F levels as significant VTE risk factors [6]. Another study from the United States in 2022, involving 223 SCD patients, pinpointed increased body mass index (BMI), prior splenectomy, and elevated white blood cell count as significant risk factors for VTE. Interestingly, neither the severity of anemia nor the hemoglobin genotype were deemed significant VTE risk factors in this study [33]. In our study, only a high WBC count and extended hospital stay emerged as statistically significant risk factors for VTE.

The pronounced incidence of VTE events in hospitalized and ambulatory SCD patients underscores the imperative need for a comprehensive evaluation of thromboprophylaxis practices tailored to this demographic. Alarmingly, there appears to be a suboptimal utilization of thromboprophylaxis, with reported usage rates oscillating between 14.4% in adolescents to 45% in adult SCD patients [34,35]. These figures are disconcertingly low, especially when juxtaposed against the inherent VTE risk in this population. The paucity of robust evidence guiding thromboprophylaxis in SCD further exacerbates this challenge. Contemporary thromboprophylaxis guidelines advocate for pharmacologic VTE prophylaxis in acutely or critically ill hospitalized adults, contingent upon an acceptable bleeding risk profile [36,37]. Regrettably, these guidelines conspicuously omit specific recommendations for SCD patients, culminating in heterogeneous clinical practices. The imminent RCT, which aims to juxtapose rivaroxaban with a placebo in SCD patients with central venous catheters (NCT05033314), is a commendable step forward. However, many such trials are indispensable to crystallize evidence-based thromboprophylaxis strategies for diverse clinical scenarios in SCD.

In our investigation, a notable 73.4% of participants were beneficiaries of some anticoagulation regimen, a proportion that markedly surpasses literature benchmarks, potentially elucidating the attenuated VTE event rates in our cohort [35,38]. The rationale for anticoagulation spans from prior VTE episodes and in-hospital thromboprophylaxis to management strategies for vaso-occlusive crises. The salutary effects of anticoagulants, particularly in ameliorating acute vaso-occlusive crises, have been empirically validated. For instance, a double-blind RCT demonstrated that tinzaparin, administered at 175 IU/kg subcutaneously daily, significantly reduced pain severity, crisis duration, and hospital stay in 127 SCD patients compared to a placebo group [39]. This evokes pertinent queries: could escalated doses of LMWH, transcending prophylactic levels, be more efficacious in SCD patients by concurrently offering anticoagulant and anti-inflammatory benefits [40]? Might the vasodilatory effects of nitric acid production, linked with LMWH use, be advantageous [41]? These conjectures warrant rigorous scientific scrutiny, especially in the context of vaso-occlusive events.

Approximately a quarter of the study participants were devoid of prophylactic anticoagulation, a phenomenon potentially exacerbated by the omission of SCD from conventional VTE risk stratification tools. This could inadvertently sideline younger patients who, despite their age, harbor significant VTE risk solely attributable to SCD. Encouragingly, there seems to be an uptrend in prophylaxis adoption over time, even among younger cohorts [35]. A recent evaluation of SCD patients with central venous access devices unveiled a decline in VTE events among those on thromboprophylaxis. Yet, the diverse anticoagulation strategies employed by clinicians preclude definitive conclusions regarding the optimal approach [42].

This study’s robustness emanates from its focus on VTE risk in hospitalized patients within a region with a high SCD prevalence, offering invaluable insights pertinent to regional ethnicities and medical practices. However, its retrospective design and reliance on health information systems introduce potential limitations, including data incompleteness for certain patient details.

Given this intrinsically vulnerable cohort’s profound morbidity and mortality ramifications, the imperative of astutely recognizing and adeptly managing VTE events in SCD patients cannot be overstated. This urgency is accentuated during hospitalizations, where concomitant VTE risk factors amplify the thrombotic threat. A meticulous approach encompassing thromboprophylaxis, early mobilization, and graduated compression stockings is paramount. This domain beckons rigorously designed randomized controlled trials, which promise transformative clinical impact.

## 5. Limitation of the Study

The retrospective nature of the study carries the potential missing of some of the patients’ data and the presence of confounders. Well-conducted prospective studies could have significant clinical importance in understanding the VTE events with better clarity.

## 6. Conclusions

Sickle cell disease inherently predisposes patients to a hypercoagulable state, amplifying their vulnerability to VTE. This susceptibility is accentuated during hospitalizations. Intriguingly, the observed VTE risk in this study was more attenuated than anticipated, potentially reflecting the efficacious thromboprophylaxis measures instituted at our institution. Our findings underscored that an elevated WBC count and prolonged hospitalization were concomitant with heightened VTE risk. Conversely, the administration of anticoagulation, irrespective of dosage, was inversely correlated with VTE occurrences. These insights must galvanize heightened clinical vigilance toward thrombotic sequelae in SCD patients. The quest for the optimal thromboprophylaxis regimen for SCD patients necessitates further rigorous research endeavors.

## Figures and Tables

**Table 1:** Details of patients’ hospitalization, type of sickle cell disease, clinical information, and VTE risk factors.

Parameter	Description	Number (%)
Gender	Male admissions	500 (47)
	Female admissions	554 (53)
Age (Mean age with range)	27.4 (13–61 years)	-
Length of stay	10.7 (1–341)	
Pregnancy	First trimester	16 (2.9)
	Second trimester	29 (5.2)
	Third trimester	42 (7.6)
	Non pregnant	467 (84.3)
Admission ward location	Medical ward	847 (80.4)
	ICU admission	27 (2.6)
	Surgical ward	63 (6.0)
	OB/GYN admission	92 (8.7)
	Not mentioned	25 (2.4)
Reason for admission	SCD with related complications	800 (75.9)
	SCD with painful crisis	655 (62.1)
	SCD with hemolytic crisis	30 (2.8)
Venous thromboembolism	SCD with pneumonia	46 (4.4)
	SCD with stroke	7 (0.7)
	SCD with cholecystitis	6 (0.6)
	VTE	7 (0.7)
	Other	254 (24.1)
Risk Factor	Surgery	62 (5.9)
	Pregnancy	87 (8.3)
	Presence of intravenous catheter	29 (2.8)
	Use of hormonal therapy	28 (2.7)
	Immobilization	11 (1.0)
	Trauma	3 (0.3)
	Other	17 (1.6)
	Total	237 (22.5)
	None	817 (77.5)
Hemoglobin Electrophoresis Result	Hb SS	364 (34.5%)
	Hb SC	5 (0.5%)
	Hb S beta0 thalassemia	190 (18.0%)
	Hb S beta+ thalassemia	102 (9.7%)
	Not done	345 (32.7%)
	Other	48 (4.6%)

OB/GYN—obstetrics or gynecology; ICU—intensive care unit; SCD—sickle cell disease; VTE—venous thromboembolism; HbSS—sickle cell anemia; HbSC—heterozygous hemoglobin S with hemoglobin C; Hb—hemoglobin.

**Table 2:** Different types of venous thrombotic events (VTE) and the treatments given.

Admission	Clinical Event	Number (%)
Presence of VTE	Yes	32 (3.0)
	No	1022 (97.0)
Type of VTE	DVT	9 (32.1)
	PE	14 (50.0)
	Cerebral Vein Thrombosis	1 (3.6)
	Other	4 (14.3)
Timing of VTE event	Admission diagnosis	7 (21.9)
	1–7 days from admission	18 (56.2)
	After day 7 post admission	7 (21.9)
**Treatment**		**Number (%)**
Antithrombotic treatment	Prophylactic LMWH	640 (60.7)
	Therapeutic LMWH	79 (7.5)
	Prophylactic Unfractionated Heparin	24 (2.3)
	Therapeutic Unfractionated Heparin	2 (0.2)
	Warfarin	33 (3.1)
	No Anticoagulation	256 (24.3)
	Other	20 (1.9)
On Hydroxyurea	Yes	443 (42.0)
	No	611 (58.0)
NSAID use	Yes	536 (50.9)
	No	518 (49.1)

VTE—venous thromboembolism, LMWH—low-molecular-weight heparin, DVT—deep vein thrombosis, PE—pulmonary embolism, NSAID—nonsteroidal anti-inflammatory drugs.

**Table 3:** Multivariate logistic regression showing association of risk factors with VTE incidence.

Parameter	Odds Ratio	Std. Error	z	*p* > |z|	*p* Value	95% CI
Use of anticoagulation (any dose)	0.404	0.153	−2.39	0.017	0.03	0.192–0.850
Length of Stay	1.013	0.005	2.48	0.013	0.020	1.002–1.023
WBC count	1.043	0.016	2.64	0.008	0.035	1.011–1.077
Any risk factor	2.983	1.114	2.93	0.003	0.003	1.434–6.205

Presence of any risk factor on the incidence of VTE included the use of antithrombotic therapy, nonsteroidal anti-inflammatory drugs (NSAIDs), and hydroxyurea (HU).

## Data Availability

The required data were obtained from the hospital information system. The data can be obtained by contacting the corresponding author and are subject to the rules and regulations of ethical standards.
